# Does the Dietary Pattern of Shanghai Residents Change across Seasons and Area of Residence: Assessing Dietary Quality Using the Chinese Diet Balance Index (DBI)

**DOI:** 10.3390/nu9030251

**Published:** 2017-03-08

**Authors:** Jiajie Zang, Huiting Yu, Zhenni Zhu, Ye Lu, Changhe Liu, Chunxia Yao, Pinqing Bai, Changyi Guo, Xiaodong Jia, Shurong Zou, Fan Wu

**Affiliations:** 1 Department of Nutrition Hygiene, Division of Health Risk Factor Monitoring and Control, Shanghai Municipal Center for Disease Control and Prevention, Shanghai 200336, China; zangjiajie@scdc.sh.cn (J.Z.); zhuzhenni@scdc.sh.cn (Z.Z.); guochangyi@scdc.sh.cn (C.G.); Jiaxiaodong@scdc.sh.cn (X.J.); zoushurong@scdc.sh.cn (S.Z.); 2 Department of Vital Statistics, Division of Health information, Shanghai Municipal Center for Disease Control and Prevention, Shanghai 200336, China; yuhuiting@scdc.sh.cn; 3 Department of Molecular Biology for Public Health, Division of Non-Communicable Diseases, Shanghai Municipal Center for Disease Control and Prevention, Shanghai 200336, China; luye@scdc.sh.cn; 4 Department of Physical Examination, Division of Non-Communicable Diseases, Shanghai Municipal Center for Disease Control and Prevention, Shanghai 200070, China; liuchanghe@scdc.sh.cn; 5 Songjiang Center for Disease Control and Prevention, Shanghai 201620, China; yaochunxia1@126.com; 6 Pudong Center for Disease Control and Prevention, Shanghai 200136, China; 340bpq@163.com

**Keywords:** Shanghai Diet and Health Survey, Diet Balance Index, season, predictors

## Abstract

Background: Few studies have applied the Chinese Diet Balance Index (DBI) in evaluating dietary quality across seasons. Method: The Shanghai Diet and Health Survey (SDHS) included 1680 participants from all districts of Shanghai from 2012 to 2013. Dietary data were obtained using three-day 24-h recall in spring, summer, fall, and winter. Higher bound score (HBS), lower bound score (LBS) and diet quality distance (DQD) were calculated according to compliance with the dietary guidelines and based on the recommendations for consumption within the main food groups. HBS, LBS, and DQD represent over-intake, under-intake, and overall imbalance of the diet, respectively. Results: 836 males and 844 females were included. The HBS indicated that 10.08%, 11.84%, 10.31%, and 12.73% people have moderate or high levels of over-intake of food in spring, summer, fall, and winter, respectively; and 74.04%, 37.61%, 53.09%, and 42.72% people have moderate or high levels of deficit food intake for each of the four seasons. The mean HBS and LBS among the four seasons were statistically significant difference (*p* < 0.001). The mean (SD) DQD was 43.27 (10.21), 35.67 (9.71), 39.19 (9.36), and 36.84 (9.45) in each season. A multivariable model showed statistically significant differences in DQD according to age, gender, occupational status, education, smoking, drinking status, season, and residency (*p* < 0.001). Conclusion: An unbalanced diet is common among people living in Shanghai. Seasonality and area of residence were found to be two significant predictors. Strengthening the accessibility and the supply of food across seasons and regions should be considered.

## 1. Introduction

Dietary intake is a modifiable lifestyle behavior that impacts quality of life and the prevalence of non-communicable diseases (NCDs), such as cardiovascular disease, type 2 diabetes and cancer [1]. Unbalanced consumption of foods high in energy (sugar, starch, and/or fat) and low in essential nutrients contributes to energy excess, becoming overweight, and obesity, while a healthy and balanced diet is the key to good nutritional status and is related to a long and healthy life.

Given that dietary intake plays a role in energy balance and has an impact on obesity and many NCDs [1,2], there has been growing interest in using indices of dietary quality to evaluate adherence to a healthy and balanced diet in different countries around the world. Several diet quality indices, usually based on established nutrient requirements and well-publicized dietary guidelines, have been developed to evaluate the healthfulness of individual diets. In the UK, the ‘Healthy Diet Score’ was developed to investigate factors affecting diet in early old age [3]. The ‘Healthy Eating Index’ was developed to evaluate dietary patterns and diet changes in the US population [4,5,6]. The “Mediterranean Diet Pattern Score” has been used to estimate population adherence to the Mediterranean dietary pattern [7,8], which has been identified as a healthy dietary pattern in Southern Italy, Greece, and Spain [9].The scores, reflecting overall diet quality, can help researchers to sort through the nutrient- and food-specific findings and provide a measure of diets that incorporate nutrient and food interactions of likely biological importance [10,11,12,13]. Moreover, the scores are easy for clinicians, dietitians, and public health doctors to use for recording people’s diet in their work setting.

In China, two dietary quality indices have been designed: the “Chinese Diet Quality Index (DQI)” [14] and the “Chinese Dietary Balance Index (DBI)”. Both indices are designed to assess under- and over-nutrition, which are important risk factors in the rise of NCDs among China’s large population going through rapid economic changes [15]. 

Historically, seasonal variations in climate affected the availability of food sources from the Paleolithic period, when diet did vary over seasons. However, results related to seasonal variations in dietary intake or availability as part of studies of modern diets have been inconsistent, with some finding evidence of seasonal variations [16,17] and some finding no seasonal variation [18,19]. With the development of modern agriculture, transportation, and food industries, the food supply is much more diverse than before and “off-season” food can be found on the table during most times of the year in developed cities like Shanghai. This phenomenon affects the dietary intake of people and their consequent health outcomes and at the same time, it is also important to understand the modern diet variation across seasons for the purposes of dietary surveillance and nutrition intervention. This study aims to evaluate dietary quality based on the DBI score across seasons, and to examine factors which may be associated with adherence to the DBI score by people in Shanghai, China.

## 2. Materials and Methods

### 2.1. Study Design

The Shanghai Diet and Health Survey (SDHS) is an ongoing open cohort which has been conducted since 2012, and has finished four survey waves in spring, summer, fall, and winter. This survey is a collaborative project among the Shanghai Municipal Center for Disease Control and Prevention, local district Centers for Disease Control and Prevention, and Community Services Centers. The SDHS was designed and implemented by the government to examine the nutritional status and food contaminants in Shanghai, and to examine how these factors affect people’s health, as rapid economic development in Shanghai has now introduced remarkable variations in diet and eating habits that may influence food intake and health outcomes.

A multistage, stratified random sampling was used to obtain a representative sample of the Shanghai population aged 15 years and over. The megacity was stratified into urban, fringe, and rural areas according to the ratio of non-agricultural registered resident counts in sub-districts to those in townships. Villages and townships within the urban, fringe, and rural areas were selected using a probability proportionate to size sampling method based on their populations. Neighborhoods were randomly selected in each village and town. Finally, 54 sites (villages and towns), including 162 neighborhoods, were randomly selected from 229 sites. A total of 1944 subjects, 15 years old and above, and their family members, were recruited into the study. Data were collected during May–June 2012 (Spring), August–September 2013 (Summer), November–December 2012 (Fall), and January–February 2013 (Winter). 

The SDHS was approved by the Ethical Review Committee of the Shanghai Municipal Center for Disease Control and Prevention. All the participants were fully informed of the purpose and the procedures of the study before enrolling and signed a written consent form. 

### 2.2. Dietary Data Collection

In the SDHS, dietary assessment is based on a combination of data collected at the individual level and a food inventory taken at the household level. To collect individual dietary data, every household member (aged 15 years or older) was asked to report all food consumed over the previous 24 h for each of three days (two working days and one weekend day) whether at home or away from home. This was defined as the three-day 24 h recall. Household condiment consumption (such as edible oils, salt, sauces, etc.) was determined by weighing all food consumed by the household over three consecutive days. Three-day 24 h recalls were done on three consecutive days to match with the weighing. It was determined by examining changes in inventory from the beginning to the end of each day, in combination with a weighing and measuring technique. All condiments remaining after the last meal before initiation of the survey were weighed and recorded. All purchases and wasted condiments were also recorded. At the end of the survey, all remaining condiments were again weighed and recorded. 

Trained field interviewers recorded the types and amounts of food consumed at each meal using food picture aids, and the location of food consumption during the previous day. The amount of food in each dish was estimated from the household inventory and the proportion of each dish consumed was reported by each person interviewed.

Data quality control was ensured by a high standard of training of the field interviewers, who were trained for at least three days in the collection of dietary data. Where outliers were found, the household and individuals in question were revisited and asked about their food consumption to resolve these discrepancies.

The food codes in the SDHS correspond with food names in the Chinese Food Composition Table, and were used for food group classification [20,21]. Total intakes (in grams) of each food group were calculated. Cooking oil and salt intake from household food consumption data were used to supplement the individual dietary data. Individual cooking oil and salt consumption was calculated according to the total amount of oil and salt consumed in the household divided by the proportions of energy consumption of individuals in the household [22]. 

### 2.3. Dietary Balance Index-07

The purpose of the Chinese DBI-07 (revised from the DBI-2002) is to enable the assessment of the overall dietary quality among the Chinese population. The DBI-07 contains seven components from the “Chinese Dietary Guideline” and the “Chinese Food Pagoda”, including: (1) cereals; (2) vegetables and fruits; (3) dairy products, soybean, and soybean products; (4) animal food; (5) condiments and alcoholic beverages; (6) dietary variety; and (7) drinking water [15]. 

The DBI-07 is further classified into 12 food subgroups and diet variety. These include: (1) rice and rice products; (2) wheat and wheat products; (3) corn, coarse grains and products, starchy roots and products; (4) dark-colored vegetables; (5) light-colored vegetables; (6) fruits; (7) soybean and soybean products; (8) milk and dairy products; (9) red meat and meat products; (10) poultry and game; (11) eggs; (12) fish and shellfish; (13) Diet variety. A score of 0 means the individual has reached or exceeded the lowest recommended intake of all of these food groups (a score of 0 is assigned for each food subgroup), while a negative score (−12 to 0 for cereals, drinking water and diet variety; −6 to 0 for vegetable, fruits, dairy, and soybean; −4 to 0 for red meat, products, poultry and game, fish, and shrimp, egg, cooking oil salt and alcoholic beverages) indicates that they did not reach the lowest recommended intake of any food group. Positive scores indicate over-intake of the recommended level of any food group. The suggested lowest intake is 5 g/day for soybean and products, and 25 g/day for the other 11 food subgroups based on Chinese DQI [14]. The details of the other DBI-07 components have been described elsewhere [15,23,24], and a brief description of each component has been provided in the results.

A score of 0 for each DBI-07 component demonstrates meeting the recommended intake amounts. The DBI-07 gives negative scores (range −12 to 0) to evaluate deficit food intake levels for vegetables and fruits; dairy products, soybean and soybean products; dietary variety; and drinking water; all of which should be consumed in “plenty” or “sufficient” amounts, as recommended by the dietary guidelines. The DBI-07 gives positive scores (range 0–12) to assess excessive food intake levels for condiments and alcoholic beverages, which the dietary guidelines recommend in “reduced” or “limited” amounts. In addition, DBI-07 gives both negative and positive scores for cereals (range −12 to 12) and animal food (range −12 to 8), which the dietary guidelines recommend consuming an “appropriate amount”. Based on the different intake amounts, individuals have different scores for each component, with the largest absolute score for each component being 12. Scoring details please find in Table S1. 

By summing scores for each DBI-07 component, we calculated three indicators of dietary quality. HBS calculates excessive food intake by adding all positive scores. LBS calculates deficit food intake by adding the absolute values of all negative scores. DQD assess unbalanced food intake by adding the absolute values of both positive and negative scores. The possible range of HBS, LBS, and DQD were: 0–32, 0–72, and 0–84, respectively [15]. The larger numbers for HBS, LBS, and DQD were indicative of poorer diet quality, whereas a score of 0 demonstrated the best diet quality [15].

For each component and indicator, a score of 0 indicates an “excellent” dietary intake (no problem); a score of less than 20% of the total score means people have a “good” dietary intake (almost no problem); between 20% and 40% of the total score means people have “acceptable” dietary intake (low level); between 40% and 60% of the total score means people have “poor” dietary intake (moderate level); and greater than 60% of the total score means people have “very poor” dietary intake (high level) [15].

### 2.4. Assessment of Other Variables

Height and weight were measured directly, by trained health workers, based on a standard protocol recommended by the World Health Organization [15]. Age groups were divided into three categories (15–44, 45–59, and >60 years). Marital status was divided into two categories (married and other marital status) based on five categories in the questionnaire. Occupation status was grouped into three levels (professional job, labor job, other). Years of education was allocated into four categories as ≤6 years, 7–9 years, 10–12 years, and >12 years. Smoking and drinking status was defined as two levels (No/Yes). Body mass index (BMI) was divided into four categorical levels based on the criteria recommended by the Working Group on Obesity in China [25], which are normal (BMI: 18.5–23.9 kg/m^2^), overweight (BMI: 24.0–27.9 kg/m^2^), obese (BMI: >28.0 kg/m^2^), and underweight (BMI: <18.5 kg/m^2^). Participants were divided into three groups according to their location (urban, suburban, and rural). Family income was classified into four levels (<20,000 RMB/person, 20,000–50,000 RMB/person, >50,000 RMB/person and not reported). Lastly, season was defined as spring, summer, fall, and winter according to the time of the survey.

### 2.5. Statistical Analysis

Mean and standard deviation (SD) were used to evaluate the average DBI scores for each component as the data were normally distributed. The chi-square test was used to test the distribution of the scores for each food group across seasons. Univariable and multivariable linear regression models were used to explore the association between DQD score and sex, age groups, marital status, occupation, education levels, smoking status, weight status, family income, and seasons. Standardized multivariable models were also conducted to assess and compare the association among each covariate. Pairwise comparisons (LSD-*t* test) were conducted to detect the differences in the DBI scores across seasons. The statistical/data analysis software package SAS 9.3 (SAS, Cary, NC, USA) was used for data analysis. 

## 3. Results

The analysis included 1680 (86.4%) sampled participants who finished the entirety of the survey for all four seasons. There were 836 males (49.76%) and 844 females (50.24%) in this study. There were similar proportions of participants aged 15–44, 45–59, and 60 or above. The characteristics of the study participants in terms of marital status, occupation, education, weight status, smoking status, drinking status, family income, and residential region are described in Table 1.

Table 2 shows the scores for the components of food intake and the percentage of Shanghai residents with each score. Excessive cereal intakes were common, with 52.78%, 43.12%, 39.72%, and 45.77% of Shanghai residents having a positive score in spring, summer, fall, and winter, respectively. The proportion of those with an over-intake of red meat products, poultry, and game was higher than that with a deficit intake all year around. In contrast, deficit intakes of vegetables and fruits, milk and soybeans, drinking water, fish and shrimps, and eggs were common, with the score for most Shanghai residents being in the negative range in all four seasons. Around 80% of people had moderate or severe levels of insufficient dairy consumption, and 46.31%, 53.64%, 50.63%, and 51.08% of Shanghai residents had a score of 0 for cooking oil in spring, summer, fall, and winter, respectively. Moreover, more than 52.04% of participants had a score of 0 for salt consumed under 6 g across the four seasons. Most of the participants drank appropriate amounts of alcoholic beverages. Dietary variety was below recommended levels, with almost all (more than 97.77%) of people in Shanghai showing negative scores. The constituent ratio of each food subgroup was statistically different among the four seasons, where summer tended to be more balanced than the other seasons for some food groups, especially fruits and vegetables (Table 2).

**Figure 1:**
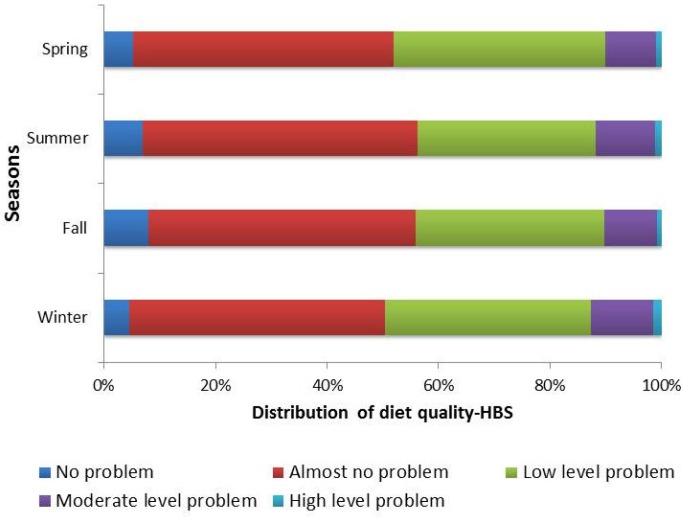
Distribution of the higher bound score (HBS) (Score range of HBS is 0–32; No problem: 0; Almost no problem: 1–6; Low level: 7–13; Moderate level: 14–19; High level: >19.

**Figure 2:**
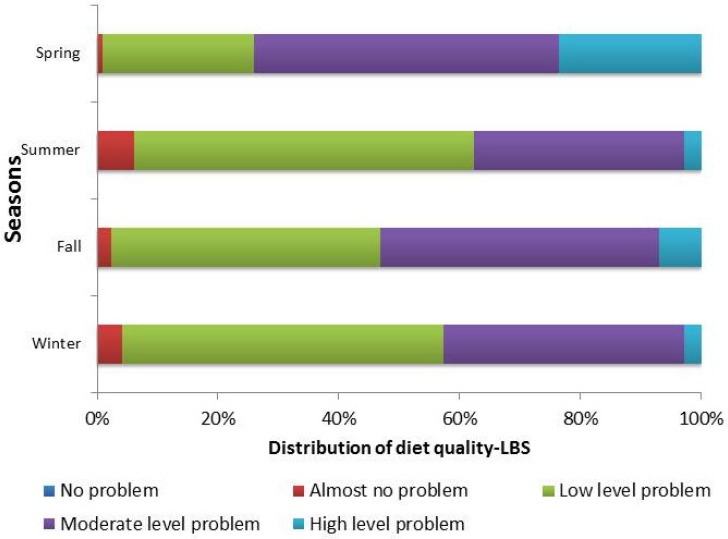
Distribution of the lower bound score (LBS) (Score range of LBS is 0–72; No problem: 0; Almost no problem: 1–14; Low level: 15–29; Moderate level: 30–43; High level: >43.

**Figure 3:**
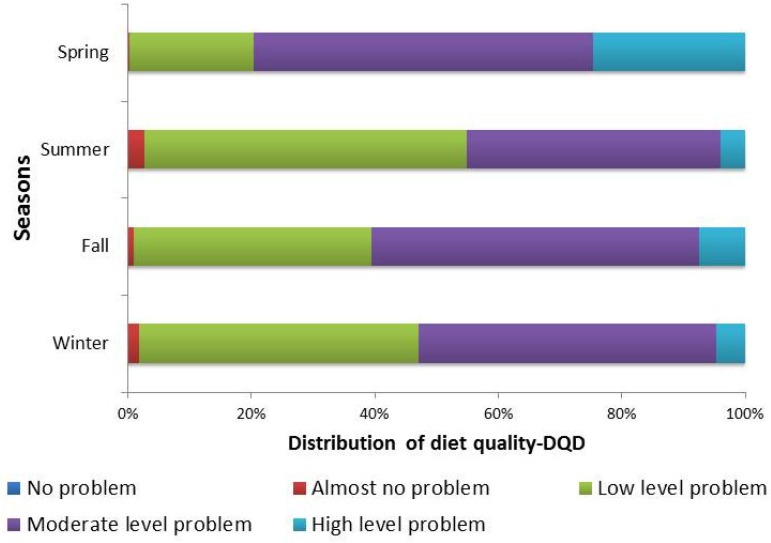
Distribution of the diet quality distance (DQD) (Score range of DQD is 0–84; No problem: 0; Almost no problem: 1–17; Low level: 18–34; Moderate level: 35–50; High level: >50.

**Figure 4:**
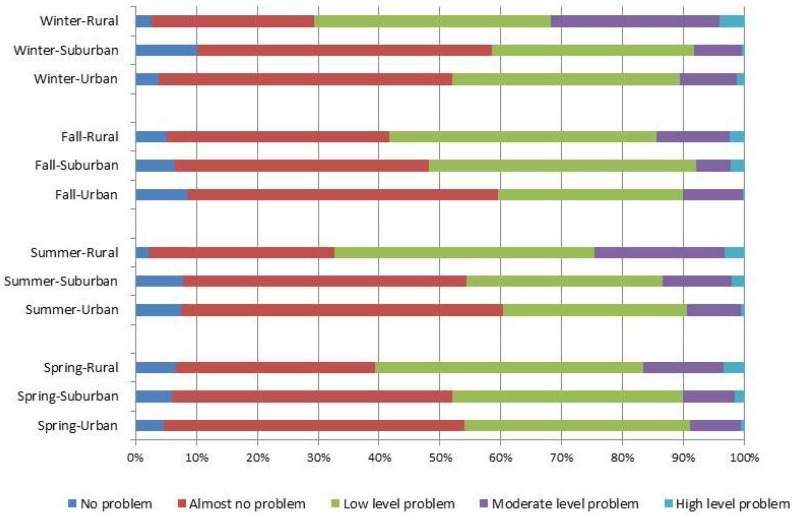
Distribution of the HBS stratified by residency regions.

**Figure 5:**
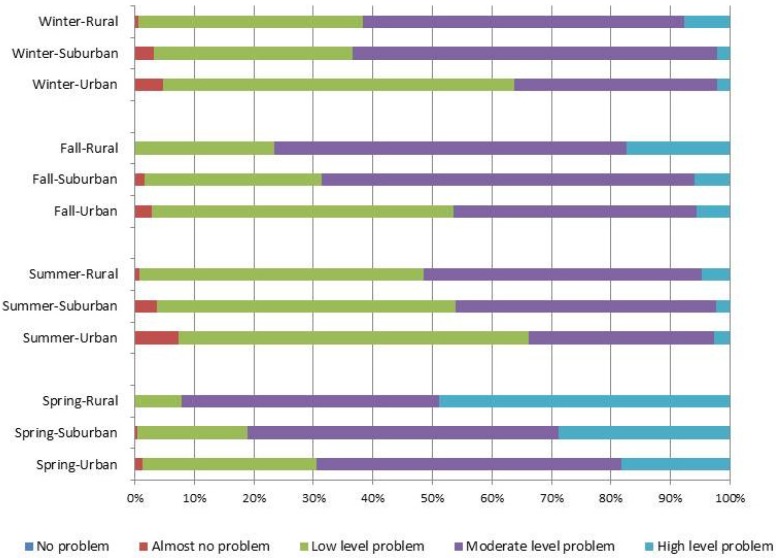
Distribution of the LBS stratified by residency regions.

**Figure 6:**
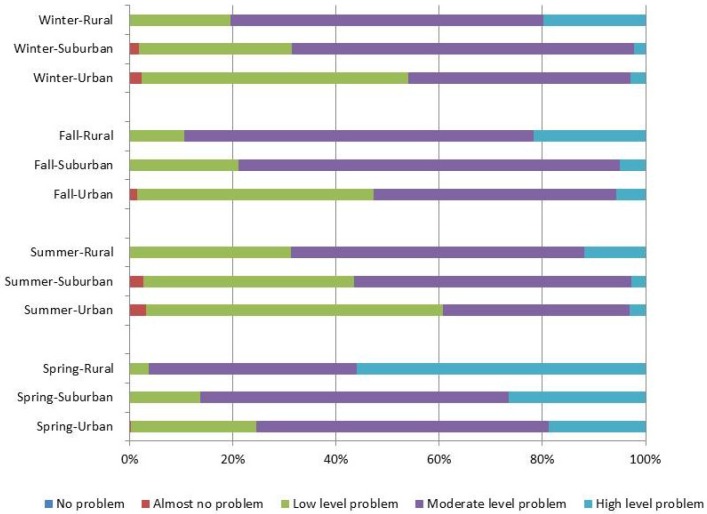
Distribution of the DQD stratified by residency regions.

The distribution of dietary quality for the HBS, LBS, and DQD are shown in Figure 1, Figure 2 and Figure 3. These scores divided by area of residence are shown in Figure 4, Figure 5 and Figure 6. The HBS indicated that 10.08%, 11.84%, 10.31%, and 12.73% of people have moderate or high levels of excessive food intake in spring, summer, fall, and winter, respectively (Figure 1). The mean of the HBS among the four seasons was statistically significantly different (*p* < 0.001) and a pairwise comparison found that fall and spring had relatively lower levels of overconsumption (Table 3).

The LBS indicates deficit food intake levels among participants: 74.04%, 37.61%, 53.09%, and 42.72% of people have moderate or high level of deficit food intake in spring, summer, fall, and winter, respectively (Figure 3). The mean of the LBS among the four seasons was statistically significantly different (*p* < 0.001) and a pairwise comparison found that summer and winter had relatively lower levels of under-consumption (Table 3).

The DQD assessed the overall imbalance in dietary intake levels, with 79.67%, 45.10%, 60.61%, and 52.95% of people having moderate or high levels of unbalanced food intake in each season (Figure 5). The mean DQD was 43.27 (10.21), 35.67 (9.71), 39.19 (9.36), and 36.84 (9.45) in spring, summer, fall, and winter, respectively. Statistically significant differences were found (*p* < 0.001) between seasons. A pairwise comparison shows that participants had the best balanced diet in summer and the worst diet in spring (Table 3).

Associations between participant characteristics and the DQD score are presented in Table 4, and the final multivariable model shows statistically significant differences in DQD according to gender (with men scoring higher than women), age (older people scored lower), occupational status (participants with a labor job and other job type had higher scores than people having professional jobs), education (more years of education had lower scores), smoking status (non-smokers had lower scores), drinking status (drinkers had higher scores), weight status (obese people had higher score than people with normal weight), regions (scored lower if living in urban rather than suburban and rural areas), family income (people with higher income had lower scores), and seasons (people scored highest in spring and lowest in summer). No significant differences were observed according to marital status.

The largest differences in scores was found among people living in different areas. The participants living in urban areas had the lowest DQD scores. However, people living in suburban and rural areas had 5.70 (95% confidence interval (CI) 5.01, 6.40) and 11.16 (95% CI, 10.50, 11.82) times higher scores. Another large difference in scores was found among seasons. The people in spring had the highest DQD score. The participants had −7.80 (95% CI: −8.59, −7.01), −4.18 (95% CI: −4.97, −3.40), and −6.69 (95% CI: −7.49, −5.90) times lower scores compared with spring to summer, fall, and winter. The standard regression model also found that area of residence and seasonality had relatively higher coefficients compared with other variables (Table 4).

## 4. Discussion

This cohort study evaluates the dietary quality of the Shanghai residents enrolled in SDHS in 2012–2013 across four seasons. The DBI-07 revealed problems with dietary quality for participants, with LBS and HBS from the DBI-07 reflecting both a deficit and a surplus of food intake, and DQD assessing the overall imbalance in dietary intake levels. We found that 45.10%–79.67% of Shanghai residents have moderate to high levels of unbalanced diets throughout the year; 10.08% to 12.73% of them had a surplus of food intake; and 37.61% to 74.04% had a deficit food intake across the four seasons. Seasonal and residential variations are main predictors for diet quality in addition to some conventional socioeconomic factors. The best diets were found in summer and worst in spring. Urban dwelling people had better diet scores than rural participants. To our knowledge, this was the first study to perform a broad analysis across seasons in regard to dietary patterns in a metropolitan area of China. 

Over-intake of cereal consumption remains a common problem, despite the nutritional transition in Shanghai and China which has seen an almost 50% reduction in cereal consumption since 1982 [26,27]. It appears that around half of the population has a surplus of cereal intake, with 10.27%–17.71% of people having extreme surplus for cereals (score 7 and above) across seasons. In addition, fine grains accounted for most of the cereals. Previous research has also shown that 68.9% of older Chinese have an over-intake of cereal, with 25.6% of older people consuming an extreme surplus of cereals according to the China Health and Nutrition Study [28]. Energy consumption has decreased with the introduction of a modern lifestyle; dietary guidelines from most countries have either recommended decreasing the total amount of cereal or increasing whole grains [29,30,31,32]. However, dietary behaviors are difficult to change in a short time. Cereals, especially fine rice and fine wheat flour, are still the staple of Shanghai residents [26]. 

Moderate to severe deficits in consumption of vegetables, fruit, milk, and soybeans were found across seasons. Although vegetable, fruit, and milk consumption have been reported as increasing during the last decade in China [26], the present study shows that the consumption is still at a low level. Previous studies the national nutrition and NCD surveys reported that Chinese people consumed 269.4 g/day and 40.7 g/day of vegetables and fruit during the period from 2010 to 2012 [32].Although the average intake of vegetables and fruit in Shanghai is shown to be 18.5 g/day and 66.3 g/day higher than the average national intake, only two thirds and half of the recommendations, respectively are being met. Our study found that the intake of vegetables and fruits appears higher in summer compared with other seasons, especially for fruits. Most fruits ripen in summer and the actual availability and accessibility of fruit in summer might be much higher than that of other seasons. Other studies have also found increases in vegetable and fruit consumption in summer compared to winter [16,17,33,34,35]. 

National nutrition and NCD surveys have also reported that Chinese people consumed 24.7 g/day of dairy on average during the period from 2010 to 2012 [32]. This consumption was still 202.8 g lower than the recommended level [31], although the population of Shanghai consumed 97.2 g of dairy per day. Various factors may have caused low dairy consumption. The prevalence of lactase deficiency among Asians is 76% to 100%, which is much higher than that among Caucasians (5%~30%) [36]. Hence, lactase intolerance is considered one of the most important reasons of the low consumption of milk in China. A previous study had shown that China’s per capita supply of dairy products is extremely low, accounting for only 3.5% of the world’s total dairy production [37]. Providing other types of dairy products instead of milk or changing the consumption style may increase dairy consumption.

Around half of the people of Shanghai had a moderate or high intake of oil and salt, which was deemed as significant contributors to the increasing prevalence of NCDs or chronic conditions [38,39]. Other studies have found no significant difference in oils or fats consumption across seasons. We found a slightly better balanced diet during summer, which might be due to the higher temperature affecting people’s appetite and cooking methods [40]. National nutrition and NCD surveys have reported that Chinese people consumed 42.1 g/day of cooking oil during the period from 2010 to 2012 [32]. In the current study, 5.7 g/day less cooking oil consumption was found. Cooking oil is the main source of fat intake for in Shanghai, which has increased approximately 50% in the last 30 years [41]. 

The consumption of salt was 7.5 g/day in this study, which was 3.0 g/day less than the average intake of the general Chinese population and 1.4 g/day less than the average intake of people in other megacities [32]. Salt consumption was lowest in summer. The main source of sodium in China is from cooking salt, which is different from Europeans and Americans where sodium mostly comes from processed food [42]. It is more important for Chinese to control sodium intake by reducing cooking salt. A mass government-led initiative on population-based salt control in Shanghai revealed that salt consumption had substantially decreased after a one-year period [43]. As studies have shown that high salt intake is associated with stroke and hypertension [38,39], effective government-led measures like this should continue to be implemented and strengthened to meet the recommendation of 6 g/day for cooking salt.

Our study found that there were statistically significant differences in diet across seasons. Summer had the most balanced diets, whereas spring had the least. Other study have reported that diet quality was significantly lower during the winter holiday, but mostly consistent by season among midlife women in Grand Forks, ND, USA. However, the study included only 52 women aged 40–60, whereas our study included 1680 representative participants, aged more than 15 years old for both genders, using a multistage random sampling method. Moreover, our study used face-to-face 24-h recall and a condiment weighting method to acquire the diet data, which is more accurate than the self-administered 24-h recall adopted in the aforementioned study [43]. The seasonal variation of food group intake in a metropolitan city like Shanghai could be due to several aspects. First, the food supply is more abundant during summer as more vegetables and fruits ripen during summer than spring. Contrary to popular belief, winter was not the worst season for a balanced diet. This is mainly due to the Chinese celebration of several important traditional and Western festivals during this time, such as Christmas and New Year, followed by the traditional spring festival and the lantern festival when people tend to consume more food as part of the festivities. Secondly, there are some traditional seasonal eating styles in China. According to theories of traditional Chinese medicine, a large number of Chinese believe in eating more meat in the winter and eating less in summer and spring in order to maintain health [44]. Additionally, people tend to consume more vegetables and fruits in the warmer seasons than during winter [45]. These factors may also lead to the variation of diet quantity and quality across seasons. Thirdly, it is of interest that we found females had better diet quality than males in summer compared with other seasons. It may be that more attention is paid to body shapes during summer than other seasons and consequently women are more likely to choose healthy diets to obtain ideal weights. 

Dietary quality is not only associated with conventional socioeconomic status [46], such as gender, occupation, and income. It is also strongly related to area of residence. People living in urban areas had lower scores than people living in suburban and rural areas. 

Firstly, food supplies and accessibility were better in urban than in rural areas. A self-supported food supply was common in rural areas where the farmers planted and consumed only certain kinds of vegetables, fruits, and grains. Our results also showed that rural people had the worst balanced diet, they consumed limited types of food, and in excess amounts. Hence, the rural people had higher levels of both over-consumption and under-consumption. Secondly, different income levels and educational backgrounds may play a role in the differences between urban and rural dwelling people [47]. A lack of nutritional knowledge, lack of cooking skills, and apathy toward nutrition messages are also potential reasons for unhealthy diets among rural people [46]. 

Previous research has shown that dietary quality is affected not only by gender, but also by education, occupation, and income level [29,46]. In our study, we also found that the DQD scores were associated with these factors. Furthermore, we found significant differences between DQD scores of younger people, smokers, drinkers, and obese people. These participants may have had less balanced scores, as they may be more likely to eat out, have fast food and take-out foods, or have less concern about individual health than their counterparts. Compared with older people (>60 years), younger (15–44) people eat out more often (6.70% vs. 66.89%). Similar results were also found between people with different smoking status (41.33% were smokers vs. 30.91% being non-smokers), drinking status (49.06% were drinkers vs. 32.38% being nondrinkers), and weight status (34.78% were of normal weight vs. 3.01% being obese) (Table S2). The results in our study were in line with other studies [30]. Future nutrition campaigns should focus on these groups, in particular the working population aged less than 45 years.

The significance of this study is that it is the first study to apply different indicators from DBI scores to a large sample of people in Shanghai, China across four seasons. We selected three indicators from DBI-07 to represent the dietary quality of our study participants. These indicators were designed for a Chinese diet. Traditional nutrition surveillance has been conducted in the fall in China since the 1950s, with researchers assuming that the diet in fall represents the diet all year round. It is important to assess the seasonal variations due to the rapid development of the economy, food manufacture, and transportation. Our study did find a seasonal difference, which provides significant evidence for clearly identifying the timing of nutritional surveillance and evaluation. The seasonal and residential variation in diet quality also shed light on the need to strengthen the food supplies across different areas and seasons to achieve equality.

One limitation of the study is that SDHS may have underestimated the surplus of oil and salt. In this study, we used the proportion of condiment intake at home to estimate the total daily intake of condiments accordingly. However, we found that Shanghai residents have dined out more often in recent years. The intake of cooking oil and salt from eating out may be higher than that from homemade food. Regarding the statistics and our models, we acknowledge a possible size effect of the sample, and even though we controlled for several potential and known cofounders, our results may still be impacted by residual confounding. We also recognize that our results should be generalized with caution to the whole Chinese population, as differences in health awareness, socioeconomic status, and lifestyles might differ significantly between the general population and our study population. Future studies could be designed to investigate actual availability and accessibility of foods to clarify the causal relationships for the main findings from this study.

## 5. Conclusions

In conclusion, an unbalanced diet is common among the Shanghai population. Some apparent imbalances include over-consumption of cereals, cooking oil, and salt, and under-intake of dairy, fruit, and vegetables. Seasonality and residency were found to be two significant and influential factors in diet quality in addition to age, gender, occupation, smoking, drinking, obesity, and income. These findings may have implications for future nutrition intervention and health education to achieve the ideal diet. Effective health promotion targeted towards specific populations should be implemented.

## Figures and Tables

**Table 1:** Characteristics of study participants.

Characteristics	Men (*n* = 836)		Women (*n* = 844)	
	*n*	% of Sub-Group	*n*	% of Sub-Group
Age group (years)				
15–44	257	30.74	253	29.98
45–59	281	33.61	292	34.60
>60	298	35.65	299	35.43
Marital Status				
Married	668	79.90	643	76.18
Other marital status	168	20.10	201	23.82
Occupation				
Professional job	215	25.72	144	17.06
Labor job	103	12.32	75	8.89
Others	518	61.96	625	74.05
Years of education				
≤6 years	170	20.33	227	26.90
7–9 years	237	28.35	254	30.09
10–12 years	215	25.72	191	22.63
>12 years	214	25.60	172	20.38
Weight Status				
Underweight	26	3.11	31	3.67
Normal	401	47.97	492	58.29
Overweight	320	38.28	258	30.57
Obese	80	9.57	35	4.15
Non-reported	9	1.08	28	3.32
Smoker				
No	424	50.72	838	99.29
Yes	412	49.28	6	0.71
Drinker				
No	500	59.81	775	91.82
Yes	275	32.89	43	5.09
Non-reported	61	7.30	26.00	3.08
Family Income				
<20,000 RMB/person	48	5.74	59	6.99
20,000–50,000 RMB/person	251	30.02	273	32.35
>50,000 RMB/person	204	24.40	204	24.17
Non-reported	333	39.83	308	36.49
Region				
Urban	350	41.87	364	43.13
Suburban	189	22.61	187	22.16
Rural	220	30.74	213	29.98
Non-reported	77	9.21	80.00	9.48

**Table 2:** Score for the components of food intake, and percentage of Shanghai residents with each score among four seasons (%).

Components	Score Range	Seasons	Score													*p-*Value *
			(−12)–(−11)	(−10)–(−9)	(−8)–(−7)	(−6)–(−5)	(−4)–(−3)	(−2)–(−1)	0	(1)–(2)	(3)–(4)	(5)–(6)	(7)–(8)	(9)–(10)	(11)–(12)	
Cereals	(−12)–(12)	Spring	0.59	1.14	1.24	3.97	8.44	16.19	15.65	18.13	14.81	7.67	5.39	2.14	4.64	<0.001
		Summer	0.67	0.39	1.62	6.17	12.06	17.53	18.43	15.36	10.85	5.72	4.42	3.28	3.49	
		Fall	1.76	1.93	3.16	6.69	12.94	17.39	16.41	13.80	10.93	6.43	4.84	2.15	1.57	
		Winter	0.35	0.98	1.70	4.33	10.71	16.17	19.99	14.60	10.57	7.97	5.21	2.45	4.97	
Vegetables	(−6)–(0)	Spring				0.25	44.14	41.96	13.65							<0.001
		Summer				0.23	33.11	47.74	18.92							
		Fall				0.71	42.63	45.16	11.50							
		Winter				0.00	36.07	49.90	14.03							
Fruits	(−6)–(0)	Spring				39.64	42.56	14.07	3.73							0.003
		Summer				23.45	26.82	23.54	26.19							
		Fall				36.41	39.38	19.82	4.39							
		Winter				34.41	39.05	21.09	5.45							
Dairy	(−6)–(0)	Spring				66.28	15.72	15.74	2.26							<0.001
		Summer				61.37	18.36	16.20	4.07							
		Fall				62.54	17.57	16.04	3.84							
		Winter				63.80	15.51	14.84	5.85							
Soybean	(−6)–(0)	Spring				42.24	14.26	9.89	33.60							<0.001
		Summer				41.30	10.58	10.18	37.95							
		Fall				35.85	12.55	14.28	37.32							
		Winter				32.42	11.39	13.29	42.91							
Red meat, products, Poultry and game	(−4)–(4)	Spring					4.02	10.84	33.34	31.29	20.51					<0.001
		Summer					4.31	5.38	32.49	29.40	28.42					
		Fall					2.56	6.04	30.40	30.60	30.41					
		Winter					1.88	4.24	26.31	30.17	37.42					
Fish and shrimp	(−4)–(0)	Spring					41.62	30.02	28.37							<0.001
		Summer					30.60	31.41	37.99							
		Fall					33.40	31.47	35.14							
		Winter					33.25	33.95	32.81							
Egg	(−4)–(4)	Spring					13.81	25.28	33.83	16.48	10.60					<0.001
		Summer					9.21	24.42	33.53	21.08	11.75					
		Fall					15.43	28.19	30.75	18.43	7.20					
		Winter					14.77	27.86	34.08	16.44	6.85					
Cooking oil	(0)–(4)	Spring							46.31	37.95	15.74					<0.001
		Summer							53.64	34.33	12.03					
		Fall							50.63	32.14	17.24					
		Winter							51.08	33.84	15.08					
Salt	(0)–(4)	Spring							58.33	31.36	10.31					<0.001
		Summer							60.03	28.65	11.32					
		Fall							55.35	32.82	11.84					
		Winter							52.04	34.40	13.57					
Alcoholic beverage	(0)–(4)	Spring							98.22	1.60	0.18					0.3085
		Summer							98.99	0.98	0.03					
		Fall							98.58	1.42	0.00					
		Winter							98.41	1.58	0.02					
Drinking water	(−12)–(0)	Spring	28.62	18.12	17.19	13.34	10.32	4.42	7.98							<0.001
		Summer	20.71	10.94	14.43	11.68	12.76	9.59	19.89							
		Fall	22.29	18.42	16.73	14.79	9.72	6.07	11.97							
		Winter	11.31	17.17	16.55	15.64	12.33	10.03	16.97							
Diet variety	(−12)–(0)	Spring	0.05	1.70	12.82	30.66	39.17	14.98	0.62							<0.001
		Summer	0.06	1.34	6.54	26.57	41.36	21.89	2.23							
		Fall	0.69	1.14	8.93	29.49	39.44	19.67	0.65							
		Winter	0.00	0.68	8.19	22.95	44.91	21.61	1.67							

* *p*-Value for chi-square test for the proportions of the scores for each food groups among seasons.

**Table 3:** Diet quality of Shanghai residents among four seasons.

	Seasons	Indicator	Mean (SD)	Range	*p*-Value *	Mean Difference and 95% CI of Pairwise Comparison ^†^		
						Summer	Fall	Winter
Over-intake	Spring	HBS	6.96 (5.46)	0–29	<0.001	−0.53 (−0.87, −0.20)	0.17 (−0.17, 0.50)	−1.23 (−1.56, −0.89)
	Summer	HBS	7.49 (4.79)	0–26			0.70 (0.36, 1.04)	−0.69 ( −1.03, −0.35)
	Fall	HBS	6.79 (4.42)	0–28				−1.39 ( −1.73, −1.05)
	Winter	HBS	8.18 (4.93)	2–27				
Under-intake	Spring	LBS	35.98 (10.76)	2–67	<0.001	7.80 (7.06, 8.54)	3.59 (2.85, 4.32)	7.32 (6.59, 8.06)
	Summer	LBS	28.18 (8.88)	3–60			−4.21 (−4.95, −3.48)	−0.48 ( −1.21, 0.26)
	Fall	LBS	32.40 (9.16)	8–68				3.74 (3.00, 4.47)
	Winter	LBS	28.66 (8.38)	6–64				
Overall imbalance	Spring	DQD	43.27 (10.21)	15–76	<0.001	7.60 (6.77, 8.42)	4.08 (3.26, 4.91)	6.43 (5.60, 0.25)
	Summer	DQD	35.67 (9.71)	12–60			−3.51 (−4.33, −2.69)	−1.17 (−1.99, −0.34)
	Fall	DQD	39.19 (9.36)	18–68				2.34 (1.52, 3.17)
	Winter	DQD	36.84 (9.45)	22–50				

* Test for means of HBS, LBS or DQD among four seasons; ^†^ Pairwise comparison of HBS, LBS, or DQD among four seasons, e.g., –0.53 (–0.87, –0.20) denotes that the summer HBS score is 0.53 lower than the spring HBS score, and the confidence interval of the mean difference is –0.87 to –0.20.

**Table 4:** Linear regression models for DQD, classified by predictors.

Items	DQD	Univariable Model		Multivariable Model		Standardized Multivariable Model	
	Mean(SD)	Coeff. (95% CI)	*p*-Value	Coeff. (95% CI)	*p*-Value	Standard Coeff. (95% CI)	*p*-Value
**Sex**							
Men	40.00 (12.35)	Reference		Reference		Reference	
Women	37.65 (11.97)	**−2.35 (−2.97, −1.73)**	**<0.001**	**−1.09 (−1.79, −0.39)**	**0.002**	**−0.04 (−0.07,** **−0.02)**	**0.003**
**Age group (years)**							
15–44	37.98 (12.26)	Reference		Reference		Reference	
45–59	38.33 (11.90)	0.35 (−0.40, 1.10)	0.359	**−1.38 (−2.19, −0.57)**	**0.001**	**−0.05 (−0.08, −0.02)**	**0.003**
>60	39.78 (12.50)	**1.80 (1.06, 2.54)**	**<0.001**	**−1.19 (−2.07, −0.32)**	**0.008**	**−0.04 (−0.08,** **−0.01)**	**0.020**
**Marital Status**							
Married	38.86 (12.19)	Reference		Reference		Reference	
Other marital status	38.30 (12.46)	−0.57 (−1.30, 0.17)	0.130	0.06 (−0.69, 0.82)	0.866	0.00 (−0.03, 0.03)	0.998
**Occupation**							
Professional job	36.63 (12.47)	Reference		Reference		Reference	
Labor job	44.02 (11.58)	**7.39 (6.28, 8.50)**	**<0.001**	**2.53 (1.37, 3.70)**	**<0.001**	**0.06 (0.03, 0.09)**	**<0.001**
Others	38.58 (12.04)	**1.95 (1.21, 2.68)**	**<0.001**	0.45 (−0.37, 1.28)	0.279	0.02 (−0.01, 0.05)	0.211
**Years of education**							
≤6 yrs	43.27 (11.87)	Reference		Reference		Reference	
7~9 yrs	39.15 (11.76)	**−4.12 (−4.92, −3.32)**	**<0.001**	**−2.30 (−3.15, −1.45)**	**<0.001**	**−0.09 (−0.12, −0.05)**	**<0.001**
10~12 yrs	37.48 (11.77)	**−5.79 (−6.63, −4.95)**	**<0.001**	**−3.06 (−4.00, −2.12)**	**<0.001**	**−0.11 (−0.14, −0.07)**	**<0.001**
>12 yrs	34.86 (12.21)	**−8.41 (−9.26, −7.55)**	**<0.001**	**−4.51 (−5.60, −3.41)**	**<0.001**	**−0.15 (−0.19, −0.11)**	**<0.001**
**Smoker**							
No	37.90 (12.18)	Reference		Reference		Reference	
Yes	41.23 (12.10)	**1.09 (0.39, 1.79)**	**0.002**	**1.87 (1.05, 2.70)**	**<0.001**	**0.07 (0.04, 0.10)**	**<0.001**
**Drinker**							
No	38.20 (12.18)	Reference		Reference		Reference	
Yes	41.26 (12.23)	**3.06 (2.30, 3.83)**	**<0.0001**	**1.18 (0.38, 1.97)**	**0.004**	**0.04 (0.01, 0.07)**	**0.002**
**Weight Status**							
Underweight	40.07 (12.48)	1.67 (−0.01, 3.36)	0.0512	0.97 (−0.59, 2.53)	0.222	**0.01 (−0.01, 0.04)**	**0.202**
Normal	37.95 (12.38)	Reference		Reference		Reference	
Overweight	38.87 (12.16)	0.48 (−0.19, 1.15)	0.1613	−0.12 (−0.75, 0.51)	0.706	−0.01 (−0.03, 0.02)	0.620
Obese	41.04 (11.27)	**2.65 (1.34, 3.96)**	**<0.0001**	**1.16 (0.00, 2.36)**	**0.049**	**0.02 (0.00, 0.04)**	**0.050**
**Region**							
Urban	35.27 (11.71)	Reference		Reference		Reference	
Suburban	40.17 (10.40)	**5.70 (5.01, 6.40)**	**<0.001**	**4.58 (3.85, 5.30)**	**<0.001**	**0.15 (0.12, 0.17)**	**<0.001**
Rural	45.62 (10.51)	**11.16 (10.50, 11.82)**	**<0.001**	**8.64 (7.90, 9.38)**	**<0.001**	**0.31 (0.28, 0.33)**	**<0.001**
**Family Income**							
<20,000 RMB/person	41.97 (12.01)	Reference		Reference		Reference	
20,000–50,000 RMB/person	39.18 (11.95)	**−2.79 (−3.58, −1.99)**	**<0.001**	−0.22 (−1.00,0.56)	0.582	−0.01 (−0.03, 0.02)	0.614
>50,000 RMB/person	36.34 (11.87)	**−5.63 (−6.34, −4.92)**	**<0.001**	**−1.63 (−2.38, −0.89)**	**<0.001**	**−0.07 (−0.10, −0.04)**	**<0.0001**
Non–reported	35.53 (12.87)	**−6.45 (−7.74, −5.15)**	**<0.001**	**−1.58 (−2.84, −0.32)**	**0.014**	**−0.03 (−0.06, −0.01)**	**0.010**
**Season**							
Spring	43.27 (17.21)	Reference		Reference		Reference	
Summer	35.67 (9.71)	**−7.60 (−8.42, −6.77)**	**<0.001**	**−7.80 (−8.59, −7.01)**	**<0.001**	**−0.28 (−0.30, −0.25)**	**<0.001**
Fall	39.19 (9.36)	**−4.08 (−4.91, −3.26)**	**<0.001**	**−4.18 (−4.97, −3.40)**	**<0.001**	**−0.15 (−0.18, −0.12)**	**<0.001**
Winter	36.84 (9.45)	**−6.43 (−7.25, −5.60)**	**<0.001**	**−6.69 (−7.49, −5.90)**	**<0.001**	**−0.24 (−0.26, −0.21)**	**<0.001**

Values in bold: statistical significant compared to references.

## Supplementary Materials

The following are available online at http://www.mdpi.com/2072-6643/9/3/251/s1, Table S1: Components of balance index revised (DBI-07), Table S2: Percentage of people who eat at home/outside for lunch.
