# Supplementary material for: Does the Dietary Pattern of Shanghai Residents Change across Seasons and Area of Residence: Assessing Dietary Quality Using the Chinese Diet Balance Index (DBI)
Source: Nutrients. 2017 Mar 8;9(3):251. doi: 10.3390/nu9030251 (PMC5372914; doi:10.3390/nu9030251)
Supplement: Supplementary file 1 [file nutrients-09-00251-s001.docx]

Supplementary Materials: Does the Dietary Pattern of Shanghai Residents Change across Seasons and Area of Residence: Assessing Dietary Quality Using the Chinese Diet Balance Index (DBI)

Jiajie Zang, Huiting Yu, Zhenni Zhu, Ye Lu, Changhe Liu, Chunxia Yao, Pinqing Bai, Changyi Guo, Xiaodong Jia, Shurong Zou and Fan Wu

**Table S1.** Components of balance index revised (DBI-07).

| Components | Score | Subgroup | Score | Intake Range by Intake Level (kJ) | | | | | | |
| --- | --- | --- | --- | --- | --- | --- | --- | --- | --- | --- |
|  |  |  |  | **6700** | **7550** | **8350** | **9200** | **10,050** | **10,900** | **11,700** |
| C1-Cereals* | (−12)–12 | Cereals | (−12)–12 ** | 0~49 = −8  200–250 g = 0  >500 g = 12 | 0~25g = −10  225~275 g = 0  >525 g = 12 | <25 g = −12  275~325 g = 0  >575 g = 12 | <25 g = −12  275~325 g = 0  >575 g = 12 | <75 g = −12  325~375 g = 0  >625 g = 12 | −12 < 125 g = −12  375~425 g = 0  >675 g = 12 | <175 g = −12  425~475 g = 0  >725 g = 12 |
| C2-Vegetables and Fruits | (−12)–0 | Vegetable | (−6)–0 | ≥300 g = 0  150~299 g = −2  1~149 g = −4  0 g = −6 |  | ≥350 g = 0  175~249 g = −2  1~175g = −4  0 g = −6 | ≥400 g = 0  200~399 g = −2  1~199 g = −4  0 g = -6 | ≥450 g =0  225~449 g = −2  1~225 g = −4  0 g = −6 | ≥500 g = 0  250~499 g = −2  1~249 g = −4  0 g = −6 |  |
|  |  | Fruits | (−6)–0 | ≥200 g = 0  100~199 g = −2  1~99 g = −4  0 g = −6 |  | ≥300 g = 0  150~299 g = −2  1~149g = −4  0 g = −6 |  | ≥400 g = 0  200~399 g = −2  1~199 g = −4  0 g = −6 |  | ≥500 g = 0  250~499 g = −2  1~249 g = −4  0 g = −6 |
| C3-Milk and dairy products,  Soybean and soybean products | (−12)–0 | Dairy | (−6)–0 | ≥ 300 g = 0, score decreased 1 with intake amount decreased 50 g | |  | |  |  | |
|  |  | Soybean | (−6)–0 | ≥30 g = 0, 15~29 g = −2  1~14 g = −4, 0 g = −6 | | ≥40 g = 0, 20~39 g = −2  1~19 g = −4, 0 g = −6 | | | ≥50 g = 0, 25~49 g = −2  1~24 g = −4, 0 g = −6 | |
| C4-Animal food | (−12)–(−8) | Red meat,  products,  Poultry and game | (−4)–4 | 0 g = −4,  1~25 g = −2  25~75 g = 0  75~125 g = 2  >125 g over = 4 | |  | 0 g = −4  1~50 g = −2  50~100 g = 0  101~150 g = 2  >150 g = 4 |  |  |  |
|  |  | Fish,and shrimp | (−4)–0 | <20 g = −4  20~29 g = −3  30~39 g = −2  40~49 g = −1  ≥50 g = 0 |  | <30 g = −4  30~44 g = −3  45~59 g = −2  60~74 g = −1  ≥75 g = 0 |  |  | <40 g = −4  40~59 g = −3  60~79 g = −2  80~99 g = −1  ≥100 g = 0 |  |
|  |  | Egg | (−4)–4 | >75 g = 4, 51~75 g = 2, 25~50 g = 0, 1~24 g = −2, 0 g = −4 | | | | | | |
| C5-Condiments and alcoholic beverage | 0–12 | Cooking oil | 0–4 | ≤25 g = 0, 26~50 g = 2, > 50 g = 4 | | | | ≤30 g = 0, 30~60 g = 2, >60 g = 4 | | |
|  |  | Salt | 0–4 | ≤6 g = 0, 7~12 g = 2, >12 g = 4 | | | | | | |
|  |  | Alcohol Beverage | 0–4 | Male：≤25 g = 0, 26~50 g =1, 51~75g = 2, 76-100g=3, >100g=4  (25 g alcohol = 750 mL beer or 250 mL wine or 75 g liquor(<38°)or 50 g liquor(> 38°)  Female：≤15 g = 0, 15~30 g = 1, 31~45 g = 2, 46~60 g = 3, >60 g = 4  (15 g alcohol = 450 mL beer or 150 mL wine or 50 g liquor(38°) or 30 g liquor(> 38°) | | | | | | |
| C6-Diet variety | (−12)–0 | Diet variety | (−12)–0 | for consumption of greater than 25 g of foods (soybean is 5 g), otherwise score is −1 | | | | | | |
| C7-Drinking water | (−12)–0 | Drinking water | (−12)–0 | > 1200 mL = 0, <100 mL = −12  score decreased 1 with intake amount decreased 100 mL from 0 to 12 | | | | | | |

* Cereal include rice, wheat，dried legumes(exclude soybean) and tubers. Intake amount means fresh amount. Sweat potato：intake amount divided by 3；
potato：intake amount divided by 4；yam and yambean：divided by 6；** score increased (decreased) 2 with 50 g intake increased (decreased) from 0 to maximal(minimal) score.

**Table S2.** Percentage of people who eat at home/outside for lunch.

|  | **Eat at Home** | | **Eat Outside** | | **Chi-Square** | ***p*** |
| --- | --- | --- | --- | --- | --- | --- |
|  | ***n*** | **%** | ***n*** | **%** |  |  |
| Age group (years) |  |  |  |  |  |  |
| 15–44 | 148 | 33.11 | 299 | 66.89 | 406.17 | <0.0001 |
| 45–59 | 342 | 65.77 | 178 | 34.23 |  |  |
| 60– | 529 | 93.30 | 38 | 6.70 |  |  |
| Smoker |  |  |  |  |  |  |
| No | 789 | 69.09 | 353 | 30.91 | 14.20 | 0.0002 |
| Yes | 230 | 58.67 | 162 | 41.33 |  |  |
| Drinker |  |  |  |  |  |  |
| No | 825 | 67.62 | 395 | 32.38 | 15.80 | 0.0002 |
| Yes | 162 | 50.94 | 156 | 49.06 |  |  |
| Weight Status |  |  |  |  |  |  |
| Underweight | 33 | 63.46 | 19 | 36.54 | 16.52 | 0.0009 |
| Normal | 405 | 65.22 | 216 | 34.78 |  |  |
| Overweight | 283 | 62.20 | 172 | 37.80 |  |  |
| Obese | 53 | 56.99 | 40 | 43.01 |  |  |
